# Mr. Ward, on Opiate Friction

**Published:** 1801-11

**Authors:** M. Ward

**Affiliations:** Manchester


### *To the Editors of the Medical and Phyfical Journal.*

Gentlemen,

I Shall be glad if you will give the following Communications a place in the next Number of your ufeful publication. To Mr. Jenkinfon, late houfe furgeon to the Manchefter Infirmary, an apology is due for the delay, his paper having been in my poffeffion nearly two years. The fecond communication, which I have been favoured with by Mr. Boutflower, a very refpectable furgeon of confiderable experience in Salford, appears of peculiar importance, as it corroborates an idea fuggefted in my firft paper on Opiate Frictions, where I endeavoured to fhew the expediency of giving them a fair trial in Tetanus and Hydrophobia.† At length an inftance of Tetanus has occurred, in which they have been made ufe of, apparently

† See Medical and Phyfical Journal for July, 1799, p. 447—9.

with confiderable advantage; and the event having proved favourable, it will probably be the means of inducing others to have recourfe to them in tetanic affections, (and I wifh I could add, in cafes of hydrophobia) by which means alone, their efficacy or inefficacy can be fully and fairly appreciated. But I fhall refrain at prefent from farther comments, as I am preparing a few obfervations on this fubject, which, with your permiffion, I purpofe fending to be inferted in your valuable Journal. Allow me only to add, that the tincture of opium appears to be, in general, better adapted for external ufe than the powder; yet, I think particular cafes may occur, where the powder will be found preferable to the tincture; as, for inftance, where it may be neceffary to join an opiate with a portion of ung. hydrargyri, (a ufeful addition where mercury, introduced by in-unction, has a tendency to pafs off by the bowels, occafioning pain, diarrhœa, &c.) befides that the tincture does not unite fo well with the ointment, the quantity generally ufed of the latter will not imbibe enough of the former to anfwer the intended purpofe.

I remain, &c.

*Manchefter, Oct.* 2, 1781. M. WARD.

---

Case I. Communicated by Mr. Jenkinson.

" Betty Richards, ætat. 14, was admitted into the houfe on July 8, 1799, in a ftate of great debility, arifing from the ravages which a caries of the hip, affifted by the mifconduct of her mafter, had made in her conftitution. She had been troubled with diarrhœa for feveral weeks; and a few days after her admiffion, a vomiting came on. Opiates, aftringents, and aromatics were prefcribed, and adminiftered in conjunction with a proper and nutritious diet and wine, without checking either the vomiting or loofenefs; opium was likewife adminiftered, clyfter-wife, in ftarch mucilage, by which the loofenefs, but not the vomiting, was reftrained for a time, and for a time only.

" Three drachms of tincture of opium, and one of the linimentum faponis, were now ordered to be rubbed into the fhins, night and morning; and all other medicines omitted. The firft portion produced a good night, though fhe had fcarcely flept at all for the laft week; and the vomiting difappeared till towards morning, when the effects of the opium had ceafed. The fecond portion and every other, for feveral days, had the fame effect as the firft; but it was now found neceffary to increafe each portion of the tincture to half an ounce. By this plan the vomiting and diarrhœa were, the one effectually prevented vented, and the other much leffened, while food (which had, previoufly to its adoption, never remained with her for more than a few minutes) was both fought for and retained.

" Her fituation was fuch as to preclude all expectation of recovery; but her life was certainly rendered more comfortable, and her exiftence prolonged feveral days.

" JOHN JENKINSON."

*Manchester Infirmary, Nov.* 3, 1799.

---

Case II. Mr. Boutflower's Letter to Mr. Ward.

" Dear Sir,

" AS the following Cafe bears teftimony to the good effects of the Opiate Frictions, recommended by you in the 5th number of the Medical Journal, I have much pleafure in fending you the particulars of it.

" John Mortice, ætat. 13, had the misfortune to be thrown under the wheel of a rope manufactory, by which the teguments and tendons of his left leg were much lacerated, and the tibia laid bare to a confiderable extent. On being called to him immediately after the accident, I brought the edges of the wound together by means of the interrupted future, and applied a bandage. The next day he was tolerably eafy. On the 4th I removed the dreffings, and was furprifed to find the appearances not fo favourable as I could have wifhed; the lips of the wound had affumed a dark colour; there was a confiderable tenfion and fwelling of the limb, which rendered it neceffary to remove the ligatures. A common poultice was now directed to be applied, and at my next vifit I had the fatisfaction to find the fore looking better; a feparation had begun to take place of the difcoloured parts, the fwelling was leffened, and every thing indicated a favourable prognofis. Thefe flattering appearances, however, did not continue long; in a day or two he complained of a ftiffnefs and rigidity of the mufcles of the neck; he had pain when he attempted to fwallow, and the new granulations of the wound had changed from a bright to a pale colour.

" From thefe fymptoms, I did not hefitate to confider the cafe as an approaching locked jaw; I therefore directed him to take a grain of opium every fix hours; to be allowed as much wine as he could drink; and, at the fuggeftion of a friend, fpirits of turpentine were applied to the wound, with a view of exciting inflammation. On the three following days he continued to grow worfe; his jaw was ftiff, more clofed, and would only admit the edge of a tea fpoon: he could not raife his his head from the pillow without affiftance; and when done negligently, gave him violent pain, and brought on convulfive motions of the whole body, of the kind termed Opifthotonos. He fweat profufely, and the opium he had taken procured him no fleep; his pulfe, during this time, was flower than natural; no inflammation had been excited in the wound from the application of the turpentine; indeed, a ftriking infenfibility was peculiarly manifeft, as he felt no pain when it was dreffed.

"I was now induced, from the fuccefs of fome trials you had made with opiate frictions, to try their effects in this cafe. Half an ounce of laudanum, combined with two drachms of oil and the yolk of an egg, was therefore directed to be divided into two equal portions, one of which was rubbed into the thighs night and morning. No internal medicine was given under this plan except two grains of opium at bed-time. For fome days the fymptoms continued with very little change or abatement; he kept his ground, at leaft, if he did not get much; the rigidity every where remained, and had extended to the injured leg. — On the 6th or 7th day (for I do not write from notes) after the application of the liniment, he was evidently better; had flept two or three hours in the night, which he had not done fince his firft attack; the jaws were ftrikingly more feparate, and I obferved on moving him on his fide to drefs his leg, it was done with much lefs difficulty than before. From this period his recovery was gradual, and he went on fo well from day to day, that I did not confider any thing neceffary for him except nourifhments; the application of the liniment was therefore difcontinued, and his recovery trufted to the opium, which he had regularly taken at bed-time. I had very foon reafon to repent of the alteration I had made, for my patient not only made little progrefs after the frictions were laid afide, but actually appeared to be growing worfe again; I was therefore obliged to give directions for them to be re-applied, which was done with manifeft advantage, till every fymptom of Tetanus had completely vanifhed.

I am, &c.

J. J. BOUTFLOWER."

*Salford, Sept. 22, 1801.*

Memoir

